# Dual inhibition of oxidative phosphorylation and glycolysis exerts a synergistic antitumor effect on colorectal and gastric cancer by creating energy depletion and preventing metabolic switch

**DOI:** 10.1371/journal.pone.0309700

**Published:** 2024-12-12

**Authors:** Yuki Aisu, Nobu Oshima, Fuminori Hyodo, Abdelazim Elsayed Elhelaly, Akihiko Masuo, Tomoaki Okada, Shigeo Hisamori, Shigeru Tsunoda, Koya Hida, Tomonori Morimoto, Hiroyuki Miyoshi, Makoto M. Taketo, Masayuki Matsuo, Leonard M. Neckers, Yoshiharu Sakai, Kazutaka Obama

**Affiliations:** 1 Department of Surgery, Kyoto University Graduate School of Medicine, Kyoto, Japan; 2 Department of Surgery, Kobe City Medical Center General Hospital, Kobe, Japan; 3 Department of Radiology, Gifu University Hospital, Gifu, Japan; 4 Department of Radiology, Frontier Science for Imaging, Gifu University, Gifu, Japan; 5 Colon Cancer Project, Kyoto University Hospital-iACT, Kyoto University Graduate School of Medicine, Kyoto, Japan; 6 National Cancer Institute, Urologic Oncology Branch, Center for Cancer Research, NIH, Bethesda, Maryland, United States of America; 7 Department of Gastrointestinal Surgery, Osaka Red Cross Hospital, Osaka, Japan; China Medical University (Taiwan), TAIWAN

## Abstract

Pyruvate is situated at the intersection of oxidative phosphorylation (OXPHOS) and glycolysis, which are the primary energy-producing pathways in cells. Cancer therapies targeting these pathways have been previously documented, indicating that inhibiting one pathway may lead to functional compensation by the other, resulting in an insufficient antitumor effect. Thus, effective cancer treatment necessitates concurrent and comprehensive suppression of both. However, whether a metabolic switch between the metabolic pathways occurs in colorectal and gastric cancer cells and whether blocking it by inhibiting both pathways has an antitumor effect remain to be determined. In the present study, we used two small molecules, namely OXPHOS and glycolysis inhibitors, to target pyruvate metabolic pathways as a cancer treatment in these cancer cells. OXPHOS and glycolysis inhibition each augmented the other metabolic pathway *in vitro* and *in vivo*. OXPHOS inhibition alone enhanced glycolysis and showed antitumor effects on colorectal and gastric cancer cells *in vitro* and *in vivo*. Moreover, glycolysis inhibition in addition to OXPHOS inhibition blocked the metabolic switch from OXPHOS to glycolysis, causing an energy depletion and deterioration of the tumor microenvironment that synergistically enhanced the antitumor effect of OXPHOS inhibitors. In addition, using hyperpolarized ^13^C-magnetic resonance spectroscopic imaging (HP-MRSI), which enables real-time and *in vivo* monitoring of molecules containing ^13^C, we visualized how the inhibitors shifted the flux of pyruvate and how this dual inhibition in colorectal and gastric cancer mouse models altered the two pathways. Integrating dual inhibition of OXPHOS and glycolysis with HP-MRSI, this therapeutic model shows promise as a future "cancer theranostics" treatment option.

## Introduction

Pyruvate is an essential intermediate in glucose oxidation and is a critical component of cellular metabolism [1]. In cells, pyruvate plays a significant role in the production of ATP through two respiration pathways: oxidative phosphorylation (OXPHOS) and glycolysis (conversion of pyruvate to lactate). Particularly in cancer cells, these pyruvate metabolic pathways are accelerated in response to the demand for high energy consumption during rapid cell growth [1–3]. During OXPHOS, pyruvate is converted to acetyl-CoA, which enters the tricarboxylic acid cycle. During glycolysis, on the other hand, pyruvate is converted to lactate by lactate dehydrogenase (LDH) A. Hereafter, the metabolic pathway converting pyruvate to lactate via LDHA will be designated as the “P-L pathway” in this manuscript (S1 Fig). An enhancement of the P-L pathway has been classically considered a feature of cancer metabolism because the P-L pathway is a more rapid metabolic pathway than OXPHOS for producing ATP [1]. However, OXPHOS has recently been indicated as an important metabolic pathway for cancer cells as well [3–5]. This is because OXPHOS has a higher energy production efficiency per glucose unit than the P-L pathway. In addition to ATP production, these two pathways support cancer cell proliferation during cell growth through complex interactions involving pH and redox balance; both of them play crucial roles in cancer cell proliferation and survival [3,6–8]. Thus, targeting the pathways is a promising therapeutic option for cancer treatment, and, to date, several attempts have been made to develop a treatment targeting them in cancer cells [9–14].

OXPHOS and glycolysis functionally complement each other according to the oxygen level in normal cells. It has been well documented that cancer cells exhibit increased lactate production regardless of the oxygen level compared to normal cells [15]. However, whether there is a metabolic interaction that could confer metabolic plasticity (henceforth referred to as "metabolic switch") in cancer cells remains unclear. This potential metabolic switch may be a compensatory mechanism to maintain energy production in cancer cells, and targeting such a metabolic switch may be the key to the therapeutic efficacy of dual inhibition. Thus far, almost all previous reports have focused on inhibiting only one pathway using small molecule inhibitors [11,16–18] and not on the metabolic flux change in pyruvate metabolism. Consequently, the potential of using chemical compounds to inhibit both metabolic pathways as a cancer treatment is of significant interest. In colorectal and gastric cancers, a metabolic switch may occur between the two pathways [9,19], adequate inhibition of both pathways may be a novel and promising treatment. In order to validate the effectiveness of this strategy, it is essential to evaluate changes in the metabolic switch. Based on the current findings, we hypothesized that the simultaneous administration of IACS-010759, a specific mitochondrial complex I inhibitor that showed OXPHOS inhibition [11], and NCI-006, a specific LDH inhibitor [10,20], would inhibit both pathways and abrogate the metabolic switch, resulting in a synergistic antitumor effect on colorectal and gastric cancer due to energy depletion.

Considering treatment using these inhibitors against colorectal and gastric cancers, it should be noted that *in vitro* metabolic profiling does not always predict *in vivo* cancer metabolism. Therefore, a noninvasive imaging approach that can dynamically monitor pyruvate metabolic flux *in vivo* would be highly beneficial for developing metabolism-targeting cancer therapy. Hyperpolarized ^13^C-magnetic resonance spectroscopic imaging (HP-MRSI) is a novel functional imaging technique that can visualize and quantify the behavior of ^13^C-containing molecules noninvasively in real time. Moreover, with ^13^C-labeled pyruvate has been reported to be a promising technique for monitoring pyruvate metabolic flux *in vivo* [21–28]. Several clinical trials have been conducted, and the safety and efficacy of this modality in cancer patients have been demonstrated [29].

In this study, we examined the effect of a therapeutic strategy involving dual inhibition of both pyruvate metabolic pathways using small molecules and monitored the potential metabolic switch between the OXPHOS and P-L pathways in colorectal and gastric cancer cells. Moreover, we evaluated the effect of these inhibitors on these pyruvate metabolic pathways and tumor microenvironment. Finally, we assessed the potential use of HP-MRSI as a tool for monitoring the pyruvate metabolic switch *in vivo*. This study aims to provide novel insights into targeted cancer therapies and metabolic imaging techniques by investigating the dual inhibition of pyruvate metabolic pathways using HP-MRSI.

## Materials and methods

### Experimental model and subject details

#### Inhibitors of mitochondrial complex I and LDH

Mitochondrial complex I inhibitor IACS-010759 was purchased from Sigma-Aldrich (St. Louis, MO, USA). Molina et al. previously described the details of this inhibitor, including pharmacokinetic data [11]. For *in vitro* experiments, IACS-010759 was dissolved in dimethyl sulfoxide (DMSO) as a 10 mM stock solution and further diluted in PBS and culture medium. For *in vivo* use, the inhibitor was dissolved in 0.5% methylcellulose in water at a final concentration of 10 mg/mL. The inhibitor was administered via oral gavage.

The LDH inhibitor, NCI-006, was provided by the National Cancer Institute (Bethesda, MD, USA). The details of this inhibitor, including pharmacokinetic data, were previously described by Oshima et al. [9] and Rai et al. [20]. For *in vitro* use, NCI-006 was dissolved in DMSO to create a 10 mM stock solution and further diluted in PBS and culture medium. For *in vivo* use, NCI-006 was dissolved in 0.1 N NaOH/PBS, and the pH was adjusted to 7.4–7.8 with 1 N HCl. The final concentration of NCI-006 was 10 mg/mL. The inhibitor was administered intravenously. For all *in vitro* experiments, 0.01% (V/V) DMSO was used as the vehicle control. For all *in vivo* experiments, the control group received an intravenous injection of 10 μL of PBS and oral administration of 10 μL of 0.5% methylcellulose.

#### Cancer cell lines

Six human colorectal cancer cell lines (HT29, HCT116, DLD-1, SW480, SW620, and Lovo) and seven human gastric cancer cell lines (MKN1, MKN7, MKN45, MKN74, AGS, HCG27, and N87) were purchased from the American Tissue Culture Collection (Manassas, VA, USA). The cells were cultured in Roswell Park Memorial Institute 1640 medium (2.0g/L of glucose without sodium pyruvate, Corning, Manassas, VA, USA) or Dulbecco’s modified Eagle’s medium (4.5g/L of glucose without sodium pyruvate, NAKALAI TESQUE INC, Kyoto, Japan) containing 10% fetal bovine serum (Life Technologies, Carlsbad CA, USA), penicillin (100 Units/ml), and streptomycin (100 mg/mL) (FUJIFILM Wako Pure Chemical Corporation, Osaka, Japan). HCT116 is a human colorectal adenocarcinoma cell line initiated from an adult male. MKN45 is a human gastric adenocarcinoma cell line initiated from the liver metastatic site of an adult female. The HCT116 and MKN45 cells were authenticated using short-tandem repeat assays. The other cell lines were not authenticated. However, early passage cells were purchased and used in the experiments. All cell lines tested negative for Mycoplasma.

#### Patient-derived cancer spheroids

Patient-derived colon cancer (HC17T) and gastric cancer (HG2T) spheroids were provided by Kyoto University Hospital iACT (Kyoto, Japan) in April 2021. These spheroids were obtained with the approval of the Ethics Committee of the Kyoto University Graduate School and Faculty of Medicine (Approval Number: 1169 and R0915-8). Written informed consent was obtained from all patients. Patient-derived cancer spheroids were suspended in Matrigel (Corning, Corning, NY, USA). The cell-Matrigel mixture was then centrally placed in each well of a 12-well cell culture plate (30 μL/well). Following matrix material polymerization at 37°C, Patient-derived cancer spheroids were cultured using the cancer medium (S1 Table). The culture medium was changed every other day [30,31].

#### Animals and tumor models

All animal experiments were approved by the Institutional Animal Ethics and Research Committee of Kyoto University (MedKyo22159; Kyoto, Japan) and conducted in accordance with institutional guidelines. The research staff underwent training at the Institute of Laboratory Animals of Graduate School of Medicine, Kyoto University. Six-week-old female KSN/slc athymic nude mice (18–24 g) were obtained from Japan SLC (Shizuoka, Japan) and housed under specific pathogen-free conditions. Humane end points were established as follows: tumor weight >10% of body weight, weight loss >20% of body weight compared to the control group, tumors causing hinderance to eating or impairing ambulation, hunched posture, dehydration, and self-mutilation. None of the mice reached the defined endpoint criteria and died before meeting them. All necessary measures were taken to minimize suffering. Five to six mice shared a microisolator cage. The housing environment was controlled with a 12-hour day/night cycle, temperature of 24°C ± 2°C, and relative humidity of 50% ± 10%. The mice’s condition was monitored least three times a week. For all procedures, the mice were placed under deep isoflurane sedation and finally euthanized by cervical dislocation.

### Method details

#### Extracellular flux (XF) analysis and ATP production rate assay

Mitochondrial respiration and glycolysis in the cancer cells were monitored in real-time using the XF analyzer XFe96 platform (Agilent, Santa Clara, CA, USA). To ensure approximately 80% confluency of cells on the well’s surface, we determined the optimal seeding density for each cell line. The optimal cell numbers per well for each cell line was as follows: AGS: 2 × 10^4^ cells, HGC27: 1.5 × 10^4^ cells, N87: 1.5 × 10^4^ cells, MKN1: 1.5 × 10^4^ cells, MKN7: 3.0 × 10^4^ cells, MKN45: 2.0 × 10^4^ cells, MKN74: 3.0 × 10^4^ cells, HT29: 2.0 × 10^4^ cells, DLD-1: 3.0 × 10^4^ cells, SW480: 3.0 × 10^4^ cells, SW620: 4.0 × 10^4^ cells, HCT116: 2.0 × 10^4^ cells, Lovo: 4.0 × 10^4^ cells. For spheroid cultures, it was not possible to use the same normalization method due to the need for a three-dimensional culture; instead, we suspended 3.0 × 10^4^ cells in 2.5 μL of Matrigel and placed the mixture centrally in each well. Cells were cultured overnight in custom XF microplates containing the culture medium and, prior to measurement, were washed and incubated in an unbuffered assay medium (Sigma-Aldrich, St. Louis, MO, USA) in the absence of CO_2_ for 1 h at 37°C. The rates of mitochondrial respiration and glycolysis were measured using the XF Assay Kit (Agilent, Santa Clara, CA, USA) and are displayed as the Oxygen Consumption Rate (OCR) and Extracellular Acidification Rate (ECAR), respectively [32]. Notably, OCR and ECAR rates are key indicators of mitochondrial respiration and glycolysis, respectively. All measurements commenced after establishing a stable baseline, followed by the addition of NCI-006 (5 μM), IACS-010759 (2 μM), or a combination of both. The ATP production rate was assessed using the XF Real-Time ATP Rate Assay Kit (Agilent, Santa Clara, CA, USA), according to the manufacturer’s instructions. The basal OCR and ECAR were measured first in the assay for ATP production rate. The mito ATP production rate was quantified by injecting oligomycin, which inhibits mitochondrial ATP synthesis and reduces OCR. The total Proton Efflux Rate (PER) is calculated from the ECAR data and the buffer factor of the assay medium. Mitochondrial-associated acidification was accounted for by completely inhibiting mitochondrial respiration with rotenone and antimycin A, and the glyco ATP production rate was calculated from the PER data [33].

#### *In vitro* and *in vivo* cell proliferation assay

*In vitro*: In six-well plates, 2 × 10^5^ cells were seeded in 2 mL of normal growth medium for 24 h prior to treatment with IACS-010759 (1 μM), NCI-006 (1 μM), or a combination of both for 48 h. Cell viability was assessed by counting using the Countess 3 Automated Cell Counter (Thermo Fisher Scientific, Inc. Waltham, MA, USA), according to the manufacturer’s instructions.

*In vivo*: Each group consisted of six mice. A total of 5×10^6^ cells in 100 μL of PBS were injected subcutaneously into the back of each mouse. Prior to treatment, the mice were randomized into treatment (or vehicle control) groups. In HCT116 xenograft mice, IACS-010759 (20 mg/[kg•dose] body weight) was administered orally every day, and NCI-006 (40 mg/[kg•dose] body weight) was administered intravenously thrice per week for a week. An equivalent volume of PBS was administered in the control group. In MKN45 xenograft mice, IACS-010759 (20 mg/[kg•dose] body weight) was administered orally five times a week, and NCI-006 (40 mg/[kg•dose] body weight) was administered intravenously twice a week for two weeks. An equivalent volume of PBS was administered in the control group. *In vivo* proliferation assay, preliminary experiments were conducted for both HCT116 and MKN45, and different regimens were employed based on the results. The tumors were allowed to reach 100 mm^3^ in volume before initiating treatment. Tumor size was measured with calipers, and the tumor volume was estimated using the formula: 0.5 × L × W^2^ (L: length, W: width). After completion of drug administration, the mice were under observation for one week. The mice were fed a normal diet throughout the experiments. The body weights of the mice were recorded during the *In vivo* proliferation assay.

#### Monitoring the inhibitior-treatment toxicity with a blood test

Blood test: The mice were randomized into three treatment groups: the control group, post-combination treatment group, and one-week-after-treatment groups. IACS-010759 (20 mg/[kg•dose] body weight) was administered orally every day, and NCI-006 (40 mg/[kg•dose] body weight) was administered intravenously thrice per week for a week; an equivalent volume of vehicle was administered in the control group. In the control and post-combination treatment groups, mice were sacrificed 4 h following the completion of drug administration, and blood was collected from the heart for a blood test.

#### Apoptosis assay

HCT116 cells were seeded in 4 mL of normal growth medium for 24 h prior to treatment with IACS-010759 (1 μM), NCI-006 (1 μM), or a combination of both for 24 h. According to the manufacturer’s instructions, 1×10^6^ HCT116 cells/mL was stained with fluorescein isothiocyanate conjugated Annexin V and propidium iodide (Annexin V-FITC Apoptosis Detection Kit, NAKALAI TESQUE INC, Kyoto, Japan) in a volume of 100 μL. Finally, 400 μL of binding buffer was added to the cells. The mixture was analyzed using a BD FACS Aria II flow cytometer (Becton, Dickinson and Co. Franklin Lakes, NJ, USA), and the percentage of apoptosis of 5,000 cells was determined.

#### *In vitro* and *in vivo* pH assay

In six-well plates, 1×10^5^ cells were seeded in 2 mL normal growth medium and treated with IACS-010759 (1 μM), NCI-006 (1 μM), or a combination of the two for 4h. The pH of the culture supernatant was assessed in a 5% CO_2_ incubator using LAQUA twin pH-22 (HORIBA Advanced Techno Co., Ltd., Kyoto, Japan; n = 3 for each group). For *in vivo* four hours after the administration of IACS-010759 (30 mg/kg body weight, orally), NCI-006 (75 mg/kg body weight, IV; intravenously), or a combination of both, the tumor was excised and homogenized. An equivalent volume of PBS was administered as a vehicle. Subsequently, ultrapure water equivalent to twice the weight of the tumor was added, and the pH of the resulting solution was quantified (n = 3 for each group).

#### Bioluminescence imaging of reactive oxygen species (ROS)

ROS detection was performed using ROS Brite 700 nm dye (AAT Bioquest, Sunnyvale, CA, USA), according to the manufacturer’s guidelines. The dye was administered intravenously at a final concentration of 10 μg/g body weight 20 min before examination. For all procedures, the animals were placed under deep isoflurane sedation. An IVIS Lumina II (Perkin Elmer, Waltham, MA, USA) was used for *in vivo* multispectral fluorescence analysis. Four hours after administration of IACS-010759 (30 mg/kg body weight, orally), NCI-006 (75 mg/kg body weight, IV), or a combination of both, the tumors were extracted from the mice, and the total radiant efficiency of each tumor was quantified immediately (n = 3 for each group).

#### HP-MRSI using ^13^C-pyruvate

*In vivo* study: HP-MRSI was performed as previously described [23] with slight modifications. Briefly, [1-^13^C] pyruvate (23 μL, 11.4 mmol/L) containing 15 mmol/L OX063 hydroxy trityl radical and 2.5 mmol/L of gadolinium chelate ProHance (Eisai, Tokyo, Japan) were hyperpolarized using the Hypersense DNP polarizer (Oxford Instruments, Abingdon, UK). The hyperpolarized sample was rapidly dissolved in 3 mL of superheated alkaline buffer to yield a final 1-^13^C pyruvate concentration of 80 mmol/L and injected intravenously (15 μL/g body weight). HP-MRSI was performed on a 1.5T scanner (Japan Redox Ltd., Fukuoka, Japan) using a 36 mm dual ^1^H/^13^C volume coil for the detection of ^1^H and ^13^C. ^13^C MR spectra were acquired every second with a spectral width of 44 kHz, TR of 1,000 ms, and a flip angle of 10° for 80 seconds. The chemical shift values for each signal used in this study were as follows: 170.2 ppm of 1-^13^C pyruvate and 183.6 ppm of 1-^13^C lactate. Each mouse received IACS-010759 (20 mg/kg body weight, orally) 3 h before the scan or NCI-006 (40 mg/kg body weight, IV) 30 min before the scan or both agents before the scan. An equivalent volume of PBS was administered as a vehicle. The 1-^13^C-Lactate/Pyruvate (L/P) ratio was calculated from the Area Under Curve (AUC) from 0–80 sec using time-intensity data.

*Ex vivo* study: After hyperpolarization and dissolution, 250 μL of the 80 mM hyperpolarized 1-^13^C pyruvate solution was quickly mixed with 350 μL of 1:1 tumor-PBS homogenate and 50 μL of 500 mM nicotinamide adenine dinucleotide in a prewarmed NMR tube. ^13^C signal acquisition was performed for 260 seconds using the 1.4T Spinsolve 60 Carbon High-Performance Benchtop NMR equipment (Magritek, New Zealand) at a flip angle of 10° and TR of 4s.

### Statistical analysis

Data were expressed as mean ± standard error. All *in vitro* experiments were performed in triplicate. Statistical analyses were conducted using the JMP Pro 16 software (SAS Institute Inc. Cary, NC, USA). Student’s *t*-test was used to compare continuous variables. Tukey’s test (two-way analysis of variance [ANOVA]) was used for multiple comparisons. All analyses were two-sided, and differences were considered statistically significant at p < 0.05.

## Results

### Effect of the inhibitors on OXPHOS and glycolysis in colorectal and gastric cancer cell lines

**Fig 1:**
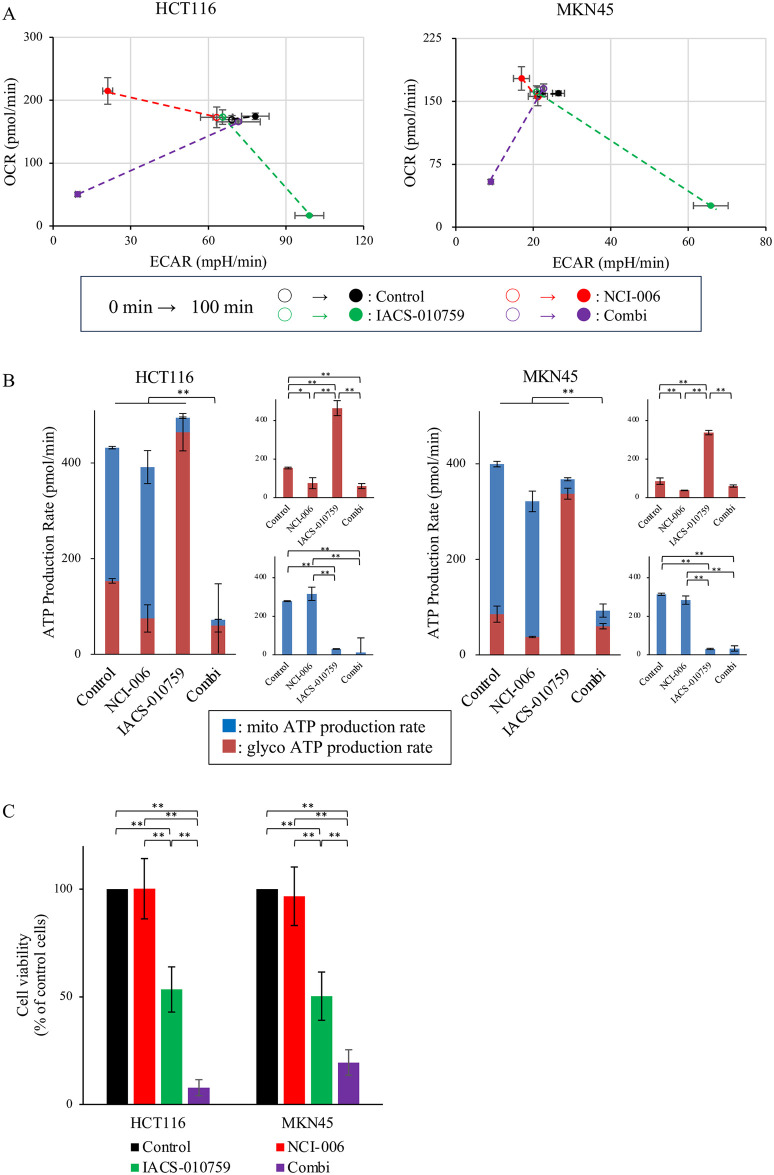
Metabolic and antitumor effect of the LDH inhibitor NCI-006 and the mitochondrial complex I inhibitor IACS-010759 in HCT116 and MKN45 cells. (A)The metabolic activities in response to treatment were determined according to the OCR/ECAR levels of HCT116 and MKN45 cells with or without treatment. Dashed lines connect the baseline activity (0 min; open symbols) and metabolic activity after treatment (100 min; closed symbols); IACS-010759 at 2 μM and/or NCI-006 at 5 μM were applied. Data are displayed as the mean ± SD. (B)The ATP production rate was measured using the XF Real-Time ATP Rate Assay Kit (Agilent Technologies, Santa Clara, CA, USA) according to the OCR/ECAR levels in HCT116 and MKN45 cells with or without treatment. Data are displayed as mean ± SD (*p < 0.05, **p < 0.01, two-way ANOVA Tukey test). (C)HCT116 and MKN45 cells were treated with NCI-006 (1 μM) and/ or IACS-010759 (1 μM) for 48 h, and cell proliferation was assessed. Data are displayed as means ± SD (n = 18 for each group; **p < 0.01, two-way ANOVA Tukey test). ECAR, Extracellular Acidification Rate.

To investigate the effect of the inhibitors on the balance between OXPHOS and glycolysis in colorectal and gastric cancer cells, OCR and ECAR were measured with and without drugs using an XF analyzer. In six colorectal cancer cell lines and seven gastric cancer cell lines, the OCR and ECAR under steady conditions differed, suggesting that the interdependent balance between OXPHOS and glycolysis varied among cell lines (S2A Fig). Furthermore, the OCR and ECAR were assessed in colorectal and gastric cancer cells after drug exposure to determine whether each inhibitor had an on-target effect (Fig 1A). In both cell lines, the OCR and ECAR decreased after the administration of IACS-010759 and NCI-006, respectively, suggesting that OXPHOS and glycolysis were inhibited in these cancer cells, thereby confirming the on-target effect of each inhibitor. Moreover, OCR and ECAR increased due to glycolysis and OXPHOS inhibition, respectively, suggesting that forced metabolic inhibition by the inhibitors has the potential to increase the complementary metabolic flux. Similar results were obtained in patient-derived cancer spheroids and other colorectal and gastric cancer cell lines (S2B Fig).

### ATP production rate assay using XF analyzer

ATP production relies on OXPHOS and glycolysis in cancer and normal cells. Therefore, the impact of pyruvate metabolic inhibition on ATP production in cancer cells was measured in HCT116 and MKN45 cells using an XF analyzer (Fig 1B). In the control group, the ratio of mitochondrial ATP to total ATP was higher than that of glycolytic ATP to total ATP (64.5% vs. 35.5% in HCT116, 78.6% vs. 21.4% in MKN45), indicating a greater energy reliance on OXPHOS than on glycolysis in both cell lines. Compared with the control group, the NCI-006-treated group showed a significant decrease in the glyco ATP production rate and no significant change in the mito ATP production rate or the total ATP production rate in either cell line. The IACS-010759-treated group showed a significant increase in the glyco ATP production rate, a significant decrease in the mito ATP production rate, and no significant change in the total ATP production rate when compared with the control group in either cell line. By each inhibitor treatment, the mito and glyco ATP production rates changed in accordance with OCR and ECAR, respectively (Fig 1A and 1B). In contrast, the mitochondrial ATP production rate did not display a significant change, primarily attributed to the minor increase in OCR within the NCI-006-treated group. In contrast to the single-treatment group, in the combination-treated group, the total ATP production rate was much lower than that in the other three groups (p<0.01) in both cell lines. Combined inhibition of both pathways resulted in a dramatic reduction in ATP production by 83.4% in HCT116 and 76.8% in MKN45. In these cell lines, single inhibitor treatment had no significant effect on the total ATP production rate, and the IACS-010759-treated group showed a dramatic complementary increase in the glyco ATP production rate, while the NCI-006-treated group showed no complementary increase in the mito ATP production rate. Compared with the IACS-010759-treated group, the combination-treated group showed a dramatic complementary decrease in the glyco ATP production rate. Taken together, dual inhibition had the effect of suppressing the complementary increase in the glyco ATP production rate.

Next, to examine the change in the source of ATP production due to metabolic inhibition, the ATP production rate index, which is calculated by dividing the ATP value from OXPHOS by that from glycolysis, was determined. As expected, this index decreased after OXPHOS inhibition and increased after P-L pathway inhibition in both cell lines (S2C Fig). This suggests that to maintain high energy consumption, these cancer cells can adapt to the small molecule-induced inhibition of each pathway by switching the metabolic pathways in either direction. Thus, targeting the metabolic flux switch between OXPHOS and glycolysis is considered to be a key factor in causing energy depletion in cancer cells.

These findings indicate that IACS-010759 enhanced glycolysis in these cancer cells. Considering the “Warburg effect”, a well-established feature of cancer metabolism characterized by increased glycolytic activity, this alteration may confer a survival advantage to tumors [3,7,34]. Subsequently, cell proliferation assays were performed to confirm the effects of these inhibitors on the OXPHOS and P-L pathways.

### *In vitro* antitumor effect of OXPHOS and glycolysis inhibition with small molecules

ATP production is a key energy source during cell activity in both normal and cancer cells. To examine the antitumor effect of the OXPHOS and P-L pathways inhibition by these molecules, a cell proliferation assay was performed using the colorectal and gastric cancer cell lines (Fig 1C). Compared with in the control group, no cell growth inhibition was observed in the NCI-006-treated group, whereas a significant inhibitory effect was observed in the IACS-010759- and combination-treated groups in both HCT116 and MKN45 cancer cell lines. Additionally, enhanced cell growth suppression was observed in the combination-treated group compared to that in the IACS-010759-treated group. The results for the other cell lines and patient-derived cancer spheroids are shown in S3A and S3B Fig The cell growth ratio of OXPHOS-inhibited cells was significantly suppressed in all cell lines. However, the cell growth ratio was significantly suppressed by P-L pathway inhibition in two colorectal (HT29 and DLD-1) and all six gastric cancer cell lines; no cell growth inhibition was observed in the other colorectal cancer cell lines. In brief, while OXPHOS inhibition by IACS-010759 had an antitumor effect in all colorectal and gastric cancer cells, the impact of P-L pathway inhibition on cell growth ratio differed depending on the cell line. Cells treated with a combination of IACS-010759 and NCI-006 had a significantly lower cell growth ratio than control cells and showed more significant growth suppression than cells treated with a single inhibitor. In other words, a combination of both inhibitors had a synergistic effect on cell growth suppression. In addition, the apoptosis assay showed that the combination-treated group exhibited a significantly higher percentage of cells in the late apoptotic stage than the control group (p < 0.01) (S3C Fig).

### *In vivo* antitumor effect of OXPHOS and glycolysis inhibition

**Fig 2:**
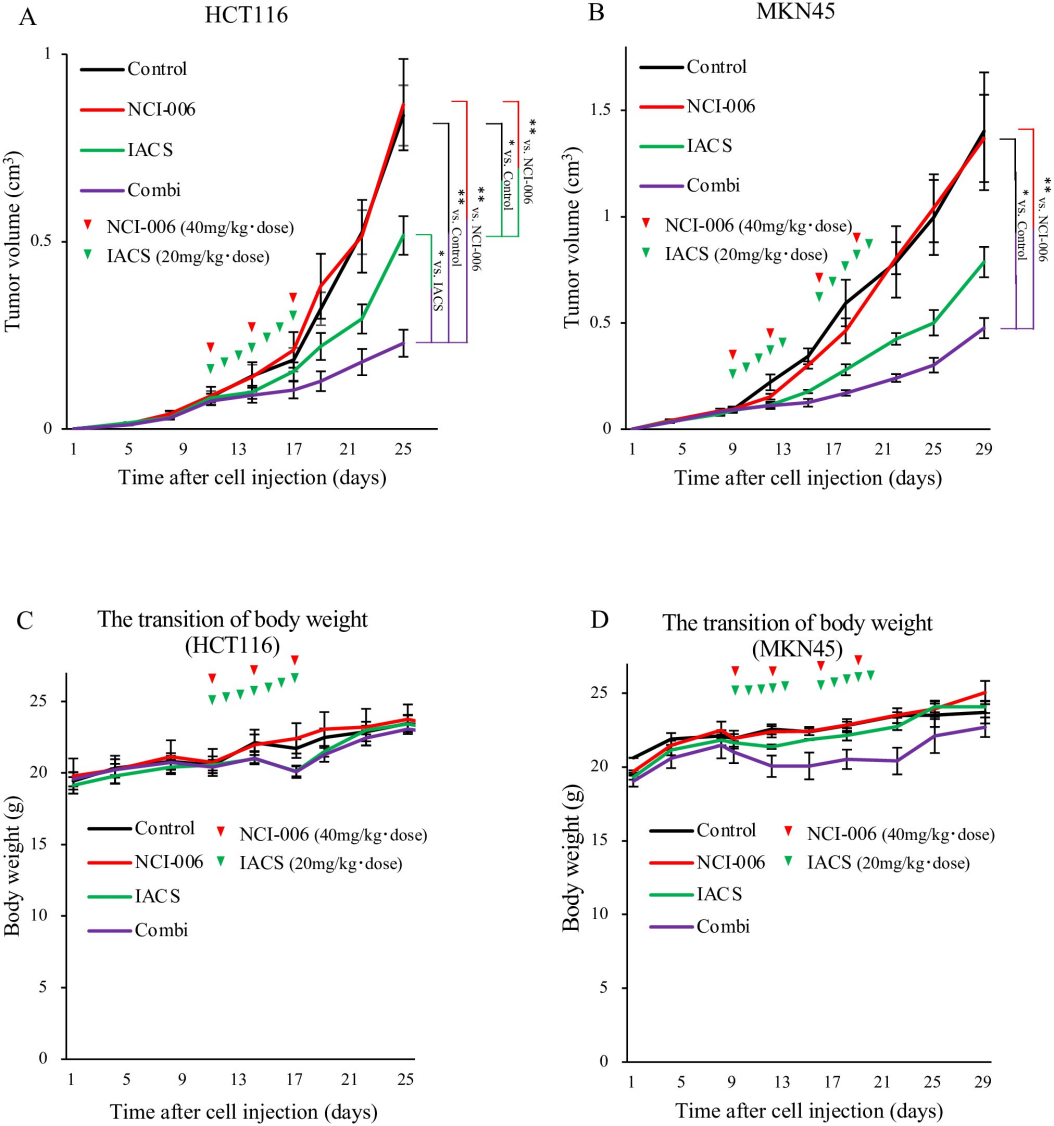
Effect of OXPHOS and glycolytic inhibition on tumor growth in HCT116 and MKN45 xenografts. (A) HCT116 cells (5×10^6^) were subcutaneously injected into the back of athymic nude. mice. After the size of the subcutaneous tumor reached 100 mm^3^, NCI-006 (40 mg/[kg•dose] body weight, red arrowheads) was administered intravenously thrice a week, and IACS-010759 (20 mg/[kg•dose] body weight, green arrowheads) was administered orally every day for a week. Data are displayed as means ± standard error of the mean (SEM) (n = 12 for each group; *p < 0.05, **p < 0.01, two-way ANOVA Tukey test). (B) MKN45 xenograft mice were generated as described in (A). NCI-006 (40. mg/[kg•dose] body weight, red arrowheads) was administered intravenously twice a week, whereas IACS-010759 (20 mg/[kg•dose] body weight, green arrowheads) was administered orally five times a week for two weeks. Data are displayed as means ± SEM (n = 6 for each group; *p < 0.05, **p < 0.01, two-way ANOVA Tukey test). (C) The body weight change of the mice in the experiment (A). (D) The body weight change of the mice in the experiment (B). Abbreviation: IACS, IACS-010759.

An *in vivo* tumor growth assay was performed using a mouse xenograft model of HCT116 and MKN45 cells. In HCT116 xenografts (Fig 2A), no inhibitory effect on tumor growth was observed in the NCI-006-treated group, whereas a significant inhibitory effect was observed in the IACS-010759 (p < 0.01) and combination-treated groups (p < 0.01) compared to in the control group. Additionally, the combination-treated group demonstrated higher suppression than the IACS-010759-treated group. In MKN45 xenografts (Fig 2B), no tumor growth inhibitory effect was observed in the NCI-006 or IACS-010759-treated groups, whereas a significant inhibitory effect was observed in the combination-treated group compared to in the control group (p < 0.01). Thus, combination treatment showed a greater capacity to suppress *in vivo* tumor growth than each single treatment.

To determine the tolerability of this inhibitor treatment, the body weight of each mouse was measured at every checkpoint. In the HCT116 experiment (Fig 2C), in the control and NCI-006-treated groups, the body weight increased consistently. The increment in body weight ceased during treatment in the IACS-010759- and combination-treated groups, and body weight promptly recovered to the level of the control group after treatment. Similarly, in the MKN45 experiment (Fig 2D), body weight gain ceased during treatment in the combination-treated groups. However, once the treatment concluded, the body weight recovered. In both HCT116 and MKN45 experiments, the average body weights of mice in all groups varied within the acceptable range of humane endpoints in the animal protocol. Besides, no fatal complications were observed in either group. We also conducted blood sampling experiments in mice to further assess the side effects of dual metabolic inhibition. Each group (n = 3) included the control group, the post-combination treatment group (following the same protocol as in Fig 2A, with blood sampling 4 h after the final dose), and the one-week-after-treatment group (with blood sampling one week after the end of the treatment using the same protocol). We performed hematological and biochemical tests, and the results are shown in S2 Table. Compared with the control group, the post-combination treatment group exhibited transiently and significantly higher levels of AST, ALT, amylase, lipase, and creatinine. In the one-week-after-treatment group, recovery was observed in all parameters. The bilirubin level was below 0.1 mg/dL in both the control and the one-week-after-treatment groups, while it transiently increased to approximately 0.3 mg/dL in the post-combination treatment group. We have previously reported that transient hemolysis occurs after administration of NCI-006 [9], and the increase in bilirubin level was considered to be an effect of the hemolysis. Together, these adverse effects were transient and reversible upon discontinuation of the treatment. From a clinical perspective, these side effects are manageable through monitoring and are considered acceptable. The therapeutic model of dual inhibition was feasible and tolerable with mice.

From these results, when P-L pathway inhibition was added to OXPHOS inhibition, the growth ratio of tumors was synergistically suppressed, whereas inhibiting P-L pathway alone did not show an antitumor effect (Fig 2A and 2B). This enhanced antitumor effect may be due to a marked decrease in ATP production or other environmental changes resulting from enhanced glycolysis. Therefore, we investigated the effects of changes on the tumor microenvironment to further understand the mechanism of additional P-L pathway inhibition.

### *In vitro* and *in vivo* pH change by the metabolic inhibitor

**Fig 3:**
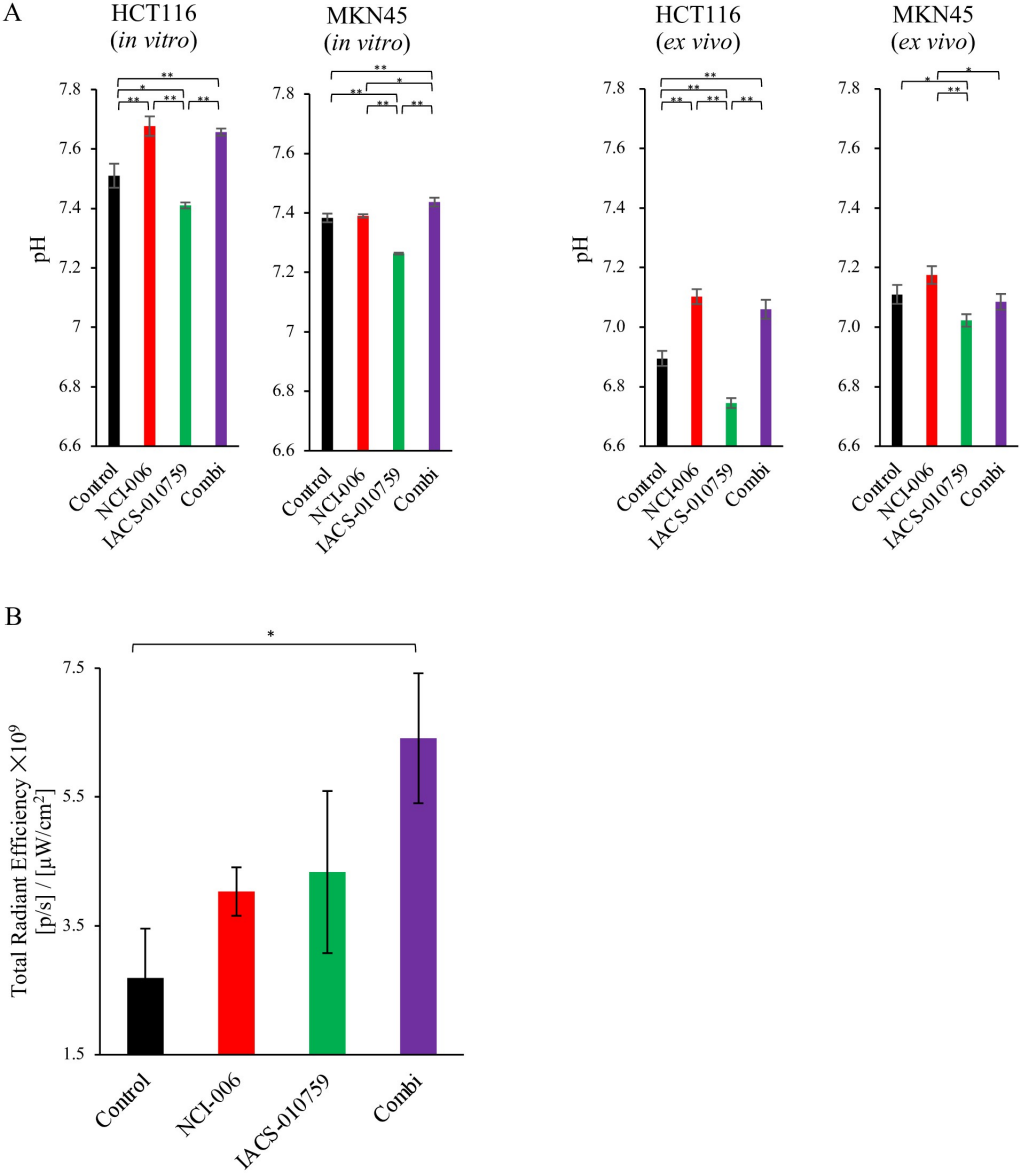
Effect of metabolic changes on tumor microenvironment. (A)The pH of the medium 4h after treatment (NCI-006 [1 μM] and/or IACS-010759 [1 μM]) was measured to evaluate pH changes *in vitro*. The pH changes *in vivo* were measured by homogenizing tumors from carcinoma-bearing mice 4 h after treatment and dilution with ultrapure water. Data are presented as means ± SEM (n = 3 for each group, *p < 0.05, **p < 0.01, *t*-test). (B)Oxidative stress in the HCT116 xenografts. Mice were injected with ROS Brite 700 nm dye 4 h after the administration of NCI-006 and/or IACS-010759, and 20 min later, the tumors were removed, and fluorescence imaging was immediately performed. Data are displayed as the means ± SEM (n = 4 for each group, *p < 0.05, *t*-test).

To examine the impact of metabolic inhibition on the tumor microenvironment, we measured the pH of the culture medium of HCT116 and MKN45 cells before and 4 h after inhibitor treatment (Fig 3A). Compared with the control group, the IACS-010759-treated group showed a significant decrease in the pH levels, indicating an increase in lactate production through the metabolic switch. Compared with that in the IACS-010759-treated group, an upward trend in pH was observed in the combination-treated group. Additionally, we measured the pH of the homogenized solution of the extracted xenograft tumors of HCT116 and MKN45 cells after inhibitor treatment. In HCT116, the pH changes observed in *ex vivo* experiments were similar to those observed *in vitro* experiments. However, in the *ex vivo* pH experiment with MKN45 cells, a slight difference was noted compared with the *in vitro* conditions. The group that received the combination treatment showed a slight decrease compared with the control group and a slight increase compared with the IACS-010759-treated group in MKN45 xenografts (Fig 3A).

### ROS production assay

Next, *in vivo* ROS production, which affects tumor growth [3,8,35], was measured before and after inhibitor treatment using ROS Brite^™^ 700. In HCT116 xenograft mice, compared with in the control group, an increasing trend in ROS production was observed in NCI-006- and IACS-010759-treated groups, and a significant increase was observed in the combination-treated group (p < 0.05, Fig 3B). Therefore, the dual inhibition induced ROS production in these cancer cells.

### Monitoring the on-target effect on the intra-tumoral OXPHOS and glycolysis *ex vivo* and *in vivo* using HP-MRSI

**Fig 4:**
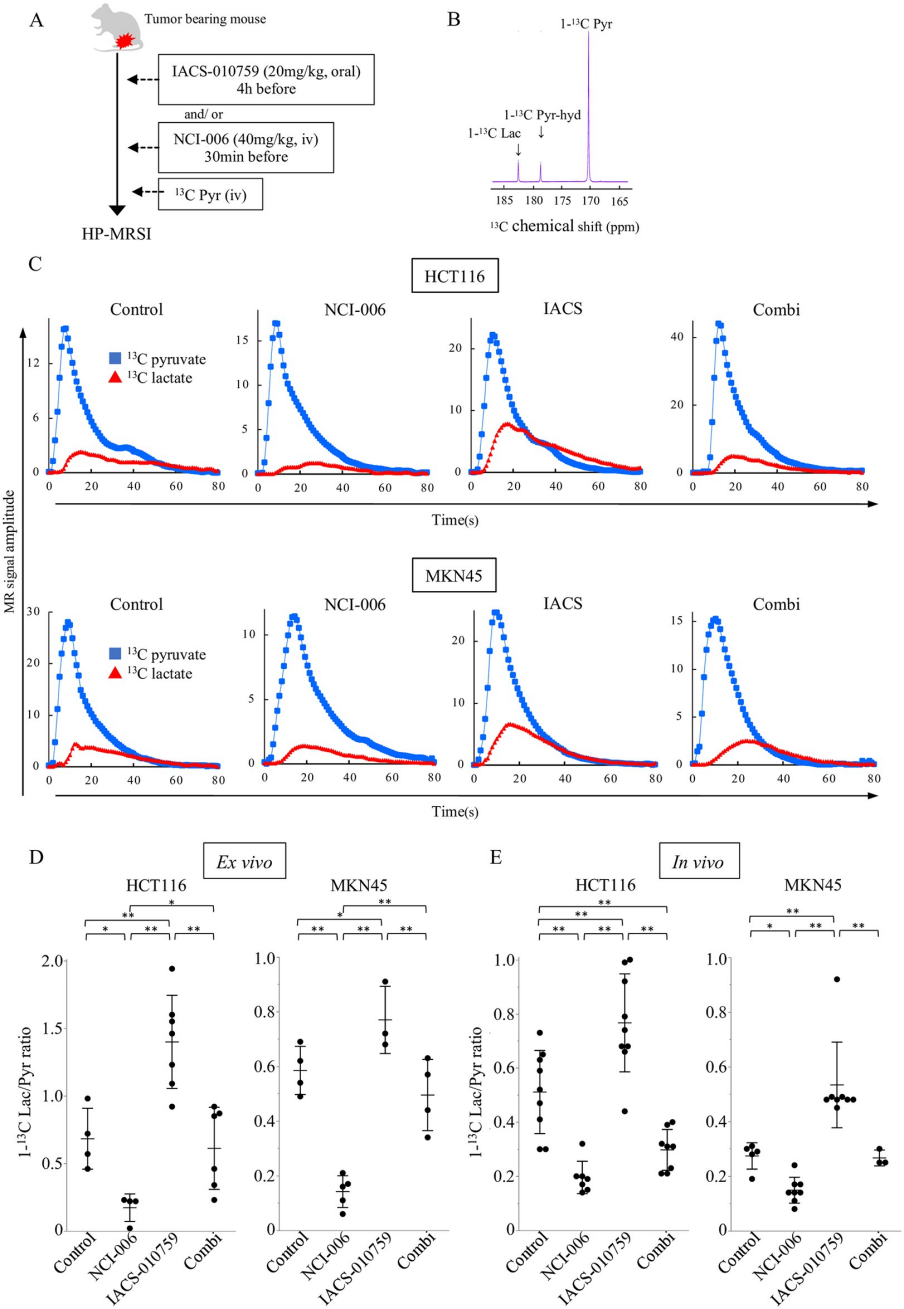
Monitoring dynamic metabolic changes in tumor xenografts using ^13^C-MRSI with hyperpolarized 1-^13^C pyruvate. (A)Schematic representation of HP 1-^13^C pyruvate MR study: ^13^C MR was performed using mice bearing HCT116 and MKN45 xenografts. Each mouse was imaged 30 minutes after NCI-006 administration and/or 3 h after IACS-010759 administration. (B)^13^C chemical shift of 1-^13^C lactate, 1-^13^C hydrate pyruvate, and 1-^13^C pyruvate. (C)Representative ^13^C-MR signal intensity curves of 1-^13^C pyruvate and 1-^13^C lactate detected in HCT116 and MKN45 xenografts after hyperpolarized 1-^13^C pyruvate injection. (D)The 1-^13^C-Lactate/Pyruvate ratio in *ex vivo* experiments. The *ex vivo* data were obtained from tumor tissue samples extracted and homogenized after each treatment. The 1-^13^C Lac/Pyr ratio was calculated from the Area Under Curve (AUC) using time-intensity data. (E)The 1-^13^C-Lactate/Pyruvate ratio *in vivo* experiments. HP-MRSI, hyperpolarized ^13^C-magnetic resonance spectroscopic imaging.

Our results confirmed that the metabolic switch between OXPHOS and glycolysis occurred i*n vitro*. To confirm whether a similar metabolic switch occurs *in vivo* in the cancer cells, we utilized HP-MRSI with hyperpolarized ^13^C-labeled pyruvate (1-^13^C pyruvate). HP-MRSI was used to monitor the *in vivo* effect of the inhibition of each metabolic pathway in HCT116 and MKN45 xenograft-bearing mice. The imaging protocol is illustrated in Fig 4A, whereas the ^13^C chemical shifts for each metabolite are illustrated in Fig 4B. We investigated the *ex vivo* and *in vivo* efficacies of oral administration of IACS-010759 and/or intravenous administration of NCI-006 on metabolic flux in each xenograft (Figs 4C and S4). In both *ex vivo* and *in vivo* experiments with HCT116 and MKN45 xenograft tumors, the ^13^C-L/P ratio was significantly lower in the NCI-006-treated group and significantly higher in the IACS-010759-treated group than in the control group (Fig 4D and 4E). NCI-006 treatment did not demonstrate tumor growth inhibitory effects in the *in vivo* proliferation assay; however, on-target effects of NCI-006 were observed in HP-MRSI. The combination-treated group had a significantly lower ^13^C-L/P ratio than the IACS-010759-treated group (p< 0.01).

## Discussion

Pyruvate metabolism, which includes OXPHOS and glycolysis, is an important energy source in cancer cells and has attracted the attention of researchers as a potential therapeutic target for several types of cancer [9–12]. Using IACS-010759, a mitochondrial complex I inhibitor for OXPHOS inhibition, and NCI-006, an LDH inhibitor for glycolytic (P-L pathway) inhibition, the current study revealed the efficacy of the dual inhibition for colorectal and gastric cancer using cancer cell lines, patient-derived cancer spheroids, and mouse xenograft models. The purpose of this study is to show the effectiveness of the combination therapy with two inhibitors. Therefore, we consider the difference between treatment regimens, as seen in the current study, is not so critical point. On the other hand, determining the best regimen for the dual inhibition model will be a future challenge. *In vitro* experiments, we also demonstrated that a bidirectional and functional shift can occur and that this shift can be blocked by dual inhibition in both colorectal and gastric cancer cells (Fig 1A). Furthermore, it is considered that the results from *in vivo* proliferation assays and the alterations in the ^13^C-L/P ratio observed by HP-MRSI following drug administrations potentially reflect the bidirectional shift and its blockage (Figs 2A, 2B and 4E).

Suppression of OXPHOS significantly increased ATP production from glycolysis, resulting in no significant change in the total ATP production rate. However, this was counteracted by dual inhibition, resulting in a sharp decrease in the total ATP production rate (Figs 1B and S2C). From the viewpoint of ATP production, the importance of glycolysis inhibition was most evident when OXPHOS was inhibited. When comparing the combination group with the control group, the glyco ATP production rate was significantly decreased in HCT116 cells but did not change noticeably in MKN45 cells (Fig 1B). Additionally, in both cell lines, the additional NCI-006 administration strongly suppressed the increase in the glyco ATP production rate induced by IACS-010759. Similarly in HP-MRSI experiments (Fig 4), in the combination group compared to the control group, the ^13^C-L/P ratio was significantly decreased in HCT116 xenografts, but there was no obvious change in MKN45 xenografts. Likewise, in both xenografts, the additional NCI-006 administration significantly suppressed the increase in the ^13^C-L/P ratio induced by IACS-010759. Taken together, one significance of glycolytic inhibition in the dual metabolic inhibition model might be the suppression of the enhanced glycolysis induced by OXPHOS inhibition.

Tumor tissues have a higher lactate production capacity and lower extracellular pH than normal tissues, suggesting that peritumoral lactate concentration and pH are related to tumor survival [34,36]. The extracellular pH, typically maintained at 7.4 in normal tissues, is often in the range of 6.5–6.9 in tumors [37]; acidic environments contribute to cancer progression [38]. Lactate accumulation in cancer cells also reduces ROS generation by decreasing reliance on mitochondrial OXPHOS and contributes to apoptosis suppression [39]. Acidosis enhances the resistance of cancer cells to glucose starvation, and conversely, improving acidosis increases sensitivity to glucose starvation and induces cell death [40]. Treatment with IACS-010759 drove glycolysis, resulting in enhanced lactate secretion and acidification of the tumor microenvironment both *in vitro* and *in vivo* (Figs 3A and 4E). Taken together, in the combination-treatment group, it is suggested that additional NCI-006 administration after IACS-010759 treatment prevented glycolysis enhancement, suppressed lactate production, and caused an increase in pH, making the tumor microenvironment further unfavorable to cancer cells. These results indicate that the combination of these two drugs suppresses tumor growth in a synergistic manner. Meanwhile, NCI-006 demonstrated the acidic tumor microenvironment reversion with no growth suppression both *in vitro* and *in vivo* in HCT116 cells. In the NCI-006-treated group, ATP production was compensated by OXPHOS, and no increase in ROS was observed, suggesting that the reversion of pH alone was insufficient to inhibit tumor growth in the cells. This finding suggests that pH is merely one of the several factors within the tumor microenvironment. Previous reports have shown that an excessive increase in intracellular ROS induces programmed cell death, such as apoptosis [8,35], and that inhibition of LDH and mitochondrial complex I via genetic methods or drugs results in an increase in intracellular ROS production [16,35,41–43]. Consistent with the previous findings, *in vivo*, tumor ROS production was significantly higher in the combination-treated group than in the control group (Fig 3B), and this seemed to have contributed to the enhanced antitumor effect. Thus, NCI-006 administration, in addition to IACS-010759, may have the capacity to not only reduce ATP production but also change the tumor microenvironment. These findings indicate that dual inhibition may be a more reasonable strategy for cancer treatment than single inhibition.

In the current study, HP-MRSI enables us to effectively monitor the inhibition of the two pyruvate metabolic pathways and subsequent bidirectional metabolic switch between OXPHOS and P-L pathway *in vivo*. Conventionally, the *in vivo* pyruvate metabolic state in tumors has been evaluated by invasive methods such as tissue sampling. However, HP-MRSI using 1-^13^C pyruvate is a noninvasive, reproducible, and objective method to evaluate pyruvate dynamics *in vivo*. This method has revealed novel and intriguing metabolic information in various organs, including several types of cancers and heart and brain disease [9,21–26,44–46].

We showed that IACS-010759 and NCI-006 reciprocally regulate the OXPHOS and P-L pathways and inhibit lactate generation from enhanced glycolysis in both *ex vivo* and *in vivo* mouse xenograft models of colorectal and gastric cancer cells (Fig 4D and 4E). We also quantified the ^13^C-L/P ratio by analyzing the HP-MRSI data with high resolution and utilized it as an indicator of the status of the two metabolic pathways derived from pyruvate in tumors. An increase in the ^13^C-L/P ratio following the IACS-010759 administration is a potential indicator of the rewiring from the OXPHOS to the P-L pathway. We have previously demonstrated the existence of this relevance by *in vivo* monitoring of ^13^C bicarbonate, a metabolite generated from the conversion from ^13^C pyruvate to acetyl-CoA (S1 Fig) [9,24]. In the current study, we, however, used the ^13^C-L/P ratio as an indicator of the metabolic switch, as visualizing the ^13^C bicarbonate signal *in vivo* with HP-MRSI is not so common owing to its originally low signal intensity [47].

The ^13^C-L/P ratio does not provide a quantitative measure of lactate production itself. However, our results show that there is still observed lactate generation, albeit to a lesser extent, in the combination treatment group (Fig 4C). While NCI-006 alone may seem to be sufficient based on the ^13^C-L/P ratio, it is crucial to recognize that inhibiting the P-L pathway alone can lead to a compensatory shift to OXPHOS, thus maintaining ATP production and enabling tumor survival (Fig 1B).

In our previous reports, NCI-006 monotherapy showed antitumor effects in the pancreatic cancer cell line MIA PaCa-2. However, in this study, we did not observe antitumor effects with NCI-006 alone in either HCT116 or MKN45 cell lines. One possible reason could be the differences in the metabolic profiles of each cancer cell line. The ^13^C-L/P ratio in MIA PaCa-2 tumors was ~0.8 and ~1.5 in magnetic resonance spectroscopic imaging and chemical shift imaging, respectively [9]. In this study, the L/P ratios in HCT116 and MKN45 tumors were 0.511 ± 0.154 and 0.274 ± 0.048, respectively, which were lower than those in MIA PaCa-2. This suggests a lower conversion efficiency from pyruvate to lactate, indicating that HCT116 and MKN45 rely less on glycolysis compared to MIA PaCa-2. The ^13^C L/P ratio determined by HP-MRSI could potentially serve as an indicator of glycolysis dependence and be useful in predicting the efficacy of NCI-006. The mechanisms in cancer cells causing this different dependence on glycolysis that clarified by HP-MRSI should be elucidated in the future study. Therefore, combination therapy targeting multiple metabolic pathways simultaneously is necessary to prevent adaptive metabolic shifts and achieve a more comprehensive inhibition of tumor metabolism. Additionally, monitoring *in vivo* metabolic fluxes with HP-MRSI is useful for determining strategies to target cancer metabolism.

Considering the clinical applications of targeting pyruvate metabolism in cancer treatment, this imaging modality should be a valuable tool for assessing biological characteristics in cancer tissues when it is impossible to directly obtain cancer tissues, especially in cases of recurrent lesions after primary tumor resection or in unresectable patients. In relation to a recent report [48] on a novel HP-MRSI probe and CD13 inhibitor capable of monitoring the on-target effect of an antitumor drug, our therapeutic model employing HP-MRSI could be extended to various other types of antitumor drugs. This extension holds the potential to assist in the identification of patients who are most likely to derive benefits from targeted treatments, thereby contributing to the advancement of personalized medicine.

In a previous study, we demonstrated that inhibiting both pathways using the same two small molecules effectively suppressed tumor growth in a pancreatic cancer model with a highly enhanced P-L pathway [9]. Here, we used colorectal and gastric cancer cell lines that are not so highly glycolytic as the pancreatic cancer cell line. Our findings newly demonstrate that these two inhibitors could inhibit the switching between the two pyruvate metabolic pathways, resulting in the deterioration of the tumor microenvironment and a synergistic increase in antitumor effects. However, the *in vivo* findings of this study are limited to single cell lines of both colorectal and gastric cancer; it is necessary to confirm these results in other cancer models or patients enrolled in clinical trials. Further, the inhibition of the two pathways is expected to not only affect cancer cells but also have adverse effects on normal tissues. In this study, we verified the tolerability *in vivo*, focusing on body weight changes and blood test; however, considering clinical application in the future, further studies should be necessary to assess the implications for normal cells and tissues.

In conclusion, this study showed that inhibition of the OXPHOS and P-L pathways led to microenvironmental deterioration and energy depletion by disrupting the bidirectional regulation of pyruvate fluxes in colorectal and gastric cancer cells, resulting in synergistic antitumor effects. HP-MRSI with 1-^13^C pyruvate was validated as a valuable imaging modality for visualizing pyruvate dynamics in this therapeutic strategy. This imaging technology allows for the monitoring of pyruvate dynamics in cancer tissue without the need for biopsy and may enable comprehensive management of the treatment process, including effective prediction and evaluation during treatment. The present treatment model of dual inhibition of oxidative phosphorylation and glycolysis using this imaging modality can be of great value to facilitate the development of novel “cancer theranostics” in the future.

## Supporting information

S1 Fig Schematic representation of OXPHOS and the P-L pathway. OXPHOS, oxidative phosphorylation; LDH, lactate dehydrogenase.
(TIF)

S2 Fig (A)The cell-energy phenotype of each cancer cell line. The cell-energy phenotype was generated based on the OCR/ECAR levels. Data are presented as mean ± SD. (B)Metabolic changes in colorectal and gastric cancer cell lines induced by each inhibitor. Metabolic activity of each cancer cell line was determined based on the OCR/ECAR levels of Lovo, MKN7, HC17T, and HG2T cells with or without treatment. Dashed lines connect the baseline activity (0 min; open symbols) and metabolic activity after treatment (100 min; closed symbols). IACS-010759 at 2 μM and/or NCI-006 at 5 μM were applied. Data are presented as mean ± SD. (C)Inhibitor-induced changes in the production source of ATP. The ATP production rate index was calculated as the ATP value from OXPHOS divided by the ATP value from glycolysis. Data are presented as the mean ± SD. OCR, oxygen consumption rate.
(TIF)

S3 Fig (A)Antitumor effects of NCI-006 and IACS-010759 in colorectal and gastric cancer cell lines. Colorectal and gastric cancer cells were treated with NCI-006 (1 μM) and/or IACS-010759 (1 μM) for 48 h, and cell proliferation was assessed. Data are displayed as mean ± SD (n = 18 for each group; **p < 0.01, two-way ANOVA Tukey test). (B)Antitumor effects of NCI-006 and IACS-010759 in patient-derived cancer spheroids from colon (HC17T) and gastric cancer (HG2T) cells. HC17T and HG2T cells were treated with NCI-006 (1 μM) and/or IACS-010759 (1 μM) for 48 h, and cell proliferation was assessed. Data are displayed as mean ± SD (n = 18 for each group; **p < 0.01, two-way ANOVA Tukey test). HC17T, patient-derived colon cancer spheroids; HG2T, patient-derived gastric cancer spheroids. (C)Flow cytometric evaluation of apoptosis in HCT116 cells. The rate of apoptotic cells was evaluated using Annexin V and propidium iodide staining and compared with vehicle and combined treatment (1 μM NCI-006 + 1 μM IACS-010759) for 24 h. Data are displayed as mean ± SEM (n = 3, ** p < 0.01).
(TIF)

S4 Fig Representative ^13^C-MR spectra of 1-^13^C pyruvate and 1-^13^C lactate detected in an HCT116 and MKN45 xenograft after hyperpolarized 1-^13^C pyruvate injection. The results correspond to Fig 4C.
(TIF)

S1 Table Culture medium of patient-derived cancer spheroids. (TIF)

S2 Table Blood test findings. (TIF)

## References

[1] Prochownik EV, Wang H. The Metabolic Fates of Pyruvate in Normal and Neoplastic Cells. Cells. 2021;10(4). doi: 10.3390/cells10040762 33808495 PMC8066905

[2] Warburg O. On the origin of cancer cells. Science. 1956;123(3191):309–14. doi: 10.1126/science.123.3191.309 13298683

[3] Martinez-Reyes I, Chandel NS. Cancer metabolism: looking forward. Nat Rev Cancer. 2021;21(10):669–80. doi: 10.1038/s41568-021-00378-6 34272515

[4] Bonekamp NA. Peter B. Hillen HS. Felser A. Bergbrede T. Choidas A., et al. Small-molecule inhibitors of human mitochondrial DNA transcription. Nature. 2020; 588, 712–6. doi: 10.1038/s41586-020-03048-z 33328633

[5] Martínez-Reyes I. Cardona LR. Kong H. Vasan K. McElroy GS. Werner M. et al. Mitochondrial ubiquinol oxidation is necessary for tumour growth. Nature. 2020; 585, 288–92. doi: 10.1038/s41586-020-2475-6 32641834 PMC7486261

[6] Corbet C, Feron O. Tumour acidosis: from the passenger to the driver’s seat. Nat Rev Cancer. 2017;17(10):577–93. doi: 10.1038/nrc.2017.77 28912578

[7] Gao Y, Zhou H, Liu G, Wu J, Yuan Y, Shang A. Tumor Microenvironment: Lactic Acid Promotes Tumor Development. J Immunol Res. 2022;2022:3119375. doi: 10.1155/2022/3119375 35733921 PMC9207018

[8] Cheung EC, Vousden KH. The role of ROS in tumour development and progression. Nat Rev Cancer. 2022;22(5):280–97. doi: 10.1038/s41568-021-00435-0 35102280

[9] Oshima N, Ishida R, Kishimoto S, Beebe K, Brender JR, Yamamoto K, et al. Dynamic Imaging of LDH Inhibition in Tumors Reveals Rapid In Vivo Metabolic Rewiring and Vulnerability to Combination Therapy. Cell Rep. 2020;30(6):1798–810.e4. 32049011 10.1016/j.celrep.2020.01.039 PMC7039685

[10] Yeung C, Gibson AE, Issaq SH, Oshima N, Baumgart JT, Edessa LD, et al. Targeting Glycolysis through Inhibition of Lactate Dehydrogenase Impairs Tumor Growth in Preclinical Models of Ewing Sarcoma. Cancer Res. 2019;79(19):5060–73. doi: 10.1158/0008-5472.CAN-19-0217 31431459 PMC6774872

[11] Molina JR, Sun Y, Protopopova M, Gera S, Bandi M, Bristow C, et al. An inhibitor of oxidative phosphorylation exploits cancer vulnerability. Nat Med. 2018;24(7):1036–46. doi: 10.1038/s41591-018-0052-4 29892070

[12] Bueno MJ, Ruiz-Sepulveda JL, Quintela-Fandino M. Mitochondrial Inhibition: a Treatment Strategy in Cancer? Curr Oncol Rep. 2021;23(4):49. doi: 10.1007/s11912-021-01033-x 33730242

[13] Ashton TM, McKenna WG, Kunz-Schughart LA, Higgins GS. Oxidative Phosphorylation as an Emerging Target in Cancer Therapy. Clin Cancer Res. 2018;24(11):2482–90. 29420223 10.1158/1078-0432.CCR-17-3070

[14] Vasan K, Werner M, Chandel NS. Mitochondrial Metabolism as a Target for Cancer Therapy. Cell Metab. 2020;32(3):341–52. doi: 10.1016/j.cmet.2020.06.019 32668195 PMC7483781

[15] Warburg O. Uber den Stoffwechsel der Karzinomezellen. Biochem Z. 1924;152:309–44.

[16] Yu H, Yin Y, Yi Y, Cheng Z, Kuang W, Li R, et al. Targeting lactate dehydrogenase A (LDHA) exerts antileukemic effects on T-cell acute lymphoblastic leukemia. Cancer Commun (Lond). 2020;40(10):501–17. doi: 10.1002/cac2.12080 32820611 PMC7571401

[17] Le A, Cooper CR, Gouw AM, Dinavahi R, Maitra A, Deck LM, et al. Inhibition of lactate dehydrogenase A induces oxidative stress and inhibits tumor progression. Proc Natl Acad Sci U S A. 2010;107(5):2037–42. doi: 10.1073/pnas.0914433107 20133848 PMC2836706

[18] Krall AS, Mullen PJ, Surjono F, Momcilovic M, Schmid EW, Halbrook CJ, et al. Asparagine couples mitochondrial respiration to ATF4 activity and tumor growth. Cell Metab. 2021;33(5):1013–26.e6. doi: 10.1016/j.cmet.2021.02.001 33609439 PMC8102379

[19] Wakiyama K, Kitajima Y, Tanaka T, Kaneki M, Yanagihara K, Aishima S, et al. Low-dose YC-1 combined with glucose and insulin selectively induces apoptosis in hypoxic gastric carcinoma cells by inhibiting anaerobic glycolysis. Sci Rep. 2017;7(1):12653. doi: 10.1038/s41598-017-12929-9 28978999 PMC5627264

[20] Rai G, Brimacombe KR, Mott BT, Urban DJ, Hu X, Yang SM, et al. Discovery and Optimization of Potent, Cell-Active Pyrazole-Based Inhibitors of Lactate Dehydrogenase (LDH). J Med Chem. 2017;60(22):9184–204. doi: 10.1021/acs.jmedchem.7b00941 29120638 PMC5894102

[21] Wang ZJ, Ohliger MA, Larson PEZ, Gordon JW, Bok RA, Slater J, et al. Hyperpolarized (13)C MRI: State of the Art and Future Directions. Radiology. 2019;291(2):273–84. doi: 10.1148/radiol.2019182391 30835184 PMC6490043

[22] Varma G, Seth P, Coutinho de Souza P, Callahan C, Pinto J, Vaidya M, et al. Visualizing the effects of lactate dehydrogenase (LDH) inhibition and LDH-A genetic ablation in breast and lung cancer with hyperpolarized pyruvate NMR. NMR Biomed. 2021;34(8):e4560. doi: 10.1002/nbm.4560 34086382 PMC8764798

[23] Saito K, Matsumoto S, Takakusagi Y, Matsuo M, Morris HD, Lizak MJ, et al. 13C-MR Spectroscopic Imaging with Hyperpolarized [1-^13^C]pyruvate Detects Early Response to Radiotherapy in SCC Tumors and HT-29 Tumors. Clin Cancer Res. 2015;21(22):5073–81. 25673698 10.1158/1078-0432.CCR-14-1717 PMC8363083

[24] Vaeggemose M, FS R, Laustsen C. Comprehensive Literature Review of Hyperpolarized Carbon-13 MRI: The Road to Clinical Application. Metabolites. 2021;11(4). doi: 10.3390/metabo11040219 33916803 PMC8067176

[25] Takakusagi Y, Kobayashi R, Saito K, Kishimoto S, Krishna MC, Murugesan R, et al. EPR and Related Magnetic Resonance Imaging Techniques in Cancer Research. Metabolites. 2023;13(1). doi: 10.3390/metabo13010069 36676994 PMC9862119

[26] Scroggins BT, Matsuo M, White AO, Saito K, Munasinghe JP, Sourbier C, et al. Hyperpolarized [1-(13)C]-Pyruvate Magnetic Resonance Spectroscopic Imaging of Prostate Cancer In Vivo Predicts Efficacy of Targeting the Warburg Effect. Clin Cancer Res. 2018;24(13):3137–48. doi: 10.1158/1078-0432.CCR-17-1957 29599412 PMC7984723

[27] Albers MJ, Bok R, Chen AP, Cunningham CH, Zierhut ML, Zhang VY, et al. Hyperpolarized ^13^C lactate, pyruvate, and alanine: noninvasive biomarkers for prostate cancer detection and grading. Cancer Res. 2008;68(20):8607–15. 18922937 10.1158/0008-5472.CAN-08-0749 PMC2829248

[28] Kurhanewicz J, Bok R, Nelson SJ, Vigneron DB. Current and potential applications of clinical ^13^C MR spectroscopy. J Nucl Med. 2008;49(3):341–4. 18322118 10.2967/jnumed.107.045112 PMC2832218

[29] Larson PE, Bernard JM, Bankson JA, Bøgh N, Bok RA, Chen AP, et al. Current Methods for Hyperpolarized [1-(13)C]pyruvate MRI Human Studies. ArXiv. 2023. doi: 10.1126/scitranslmed.3006070 38441968 PMC10997462

[30] Morimoto T, Takemura Y, Miura T, Yamamoto T, Kakizaki F, An H, et al. Novel and efficient method for culturing patient-derived gastric cancer stem cells. Cancer Sci. 2023. doi: 10.1111/cas.15840 37208931 PMC10394150

[31] Miyoshi H, Maekawa H, Kakizaki F, Yamaura T, Kawada K, Sakai Y, et al. An improved method for culturing patient-derived colorectal cancer spheroids. Oncotarget. 2018;9(31):21950–64. doi: 10.18632/oncotarget.25134 29774115 PMC5955161

[32] Dranka BP, Benavides GA, Diers AR, Giordano S, Zelickson BR, Reily C, et al. Assessing bioenergetic function in response to oxidative stress by metabolic profiling. Free Radic Biol Med. 2011;51(9):1621–35. doi: 10.1016/j.freeradbiomed.2011.08.005 21872656 PMC3548422

[33] Mookerjee SA, Gerencser AA, Nicholls DG, Brand MD. Quantifying intracellular rates of glycolytic and oxidative ATP production and consumption using extracellular flux measurements. J Biol Chem. 2017;292(17):7189–207. doi: 10.1074/jbc.M116.774471 28270511 PMC5409486

[34] de la Cruz-López KG, Castro-Muñoz LJ, Reyes-Hernández DO, García-Carrancá A, Manzo-Merino J. Lactate in the Regulation of Tumor Microenvironment and Therapeutic Approaches. Front Oncol. 2019;9:1143. doi: 10.3389/fonc.2019.01143 31737570 PMC6839026

[35] Perillo B, Di Donato M, Pezone A, Di Zazzo E, Giovannelli P, Galasso G, et al. ROS in cancer therapy: the bright side of the moon. Exp Mol Med. 2020;52(2):192–203. doi: 10.1038/s12276-020-0384-2 32060354 PMC7062874

[36] Gerweck LE, Seetharaman K. Cellular pH gradient in tumor versus normal tissue: potential exploitation for the treatment of cancer. Cancer Res. 1996;56(6):1194–8. 8640796

[37] Gerweck LE, Seetharaman K. Cellular pH gradient in tumor versus normal tissue: potential exploitation for the treatment of cancer. Cancer Res. 1996;56(6);1194–8. 8640796

[38] Corbet C. Feron O. Tumour acidosis: from the passenger to the driver’s seat. Nat Rev Cancer. 2017;17(10):577–93. 28912578 10.1038/nrc.2017.77

[39] Aykin-Burns N, Ahmad IM, Zhu Y, Oberley LW, Spitz DR. Increased levels of superoxide and H2O2 mediate the differential susceptibility of cancer cells versus normal cells to glucose deprivation. Biochem J. 2009;418(1):29–37. doi: 10.1042/BJ20081258 18937644 PMC2678564

[40] Wu H, Ding Z, Hu D, Sun F, Dai C, Xie J, et al. Central role of lactic acidosis in cancer cell resistance to glucose deprivation-induced cell death. J Pathol. 2012;227(2):189–99. doi: 10.1002/path.3978 22190257

[41] Wang ZY, Loo TY, Shen JG, Wang N, Wang DM, Yang DP, et al. LDH-A silencing suppresses breast cancer tumorigenicity through induction of oxidative stress mediated mitochondrial pathway apoptosis. Breast Cancer Res Treat. 2012;131(3):791–800. doi: 10.1007/s10549-011-1466-6 21452021

[42] Zhai X, Yang Y, Wan J, Zhu R, Wu Y. Inhibition of LDH-A by oxamate induces G2/M arrest, apoptosis and increases radiosensitivity in nasopharyngeal carcinoma cells. Oncol Rep. 2013;30(6):2983–91. doi: 10.3892/or.2013.2735 24064966

[43] Li N, Ragheb K, Lawler G, Sturgis J, Rajwa B, Melendez JA, et al. Mitochondrial complex I inhibitor rotenone induces apoptosis through enhancing mitochondrial reactive oxygen species production. J Biol Chem. 2003;278(10):8516–25. doi: 10.1074/jbc.M210432200 12496265

[44] Granlund KL, Tee SS, Vargas HA, Lyashchenko SK, Reznik E, Fine S, et al. Hyperpolarized MRI of Human Prostate Cancer Reveals Increased Lactate with Tumor Grade Driven by Monocarboxylate Transporter 1. Cell Metab. 2020;31(1):105–14 e3. doi: 10.1016/j.cmet.2019.08.024 31564440 PMC6949382

[45] Li Y, Vigneron DB, Xu D. Current human brain applications and challenges of dynamic hyperpolarized carbon-13 labeled pyruvate MR metabolic imaging. Eur J Nucl Med Mol Imaging. 2021;48(13):4225–35. doi: 10.1007/s00259-021-05508-8 34432118 PMC8566394

[46] Cunningham CH, Lau JY, Chen AP, Geraghty BJ, Perks WJ, Roifman I, et al. Hyperpolarized ^13^C Metabolic MRI of the Human Heart: Initial Experience. Circ Res. 2016;119(11):1177–82. 27635086 10.1161/CIRCRESAHA.116.309769 PMC5102279

[47] Brender JR, Kishimoto S, Merkle H, Reed G, Hurd RE, Chen AP, et al. Dynamic Imaging of Glucose and Lactate Metabolism by (13)C-MRS without Hyperpolarization. Sci Rep. 2019;9(1):3410. doi: 10.1038/s41598-019-38981-1 30833588 PMC6399318

[48] Saito Y, Yatabe H, Tamura I, Kondo Y, Ishida R, Seki T, et al. Structure-guided design enables development of a hyperpolarized molecular probe for the detection of aminopeptidase N activity in vivo. Sci Adv. 2022;8(13):eabj2667. doi: 10.1126/sciadv.abj2667 35353577 PMC8967239

