# Supplementary figures and images for: Dual inhibition of oxidative phosphorylation and glycolysis exerts a synergistic antitumor effect on colorectal and gastric cancer by creating energy depletion and preventing metabolic switch
Source: PLoS One. 2024 Dec 12;19(12):e0309700. doi: 10.1371/journal.pone.0309700 (PMC11637386; doi:10.1371/journal.pone.0309700)

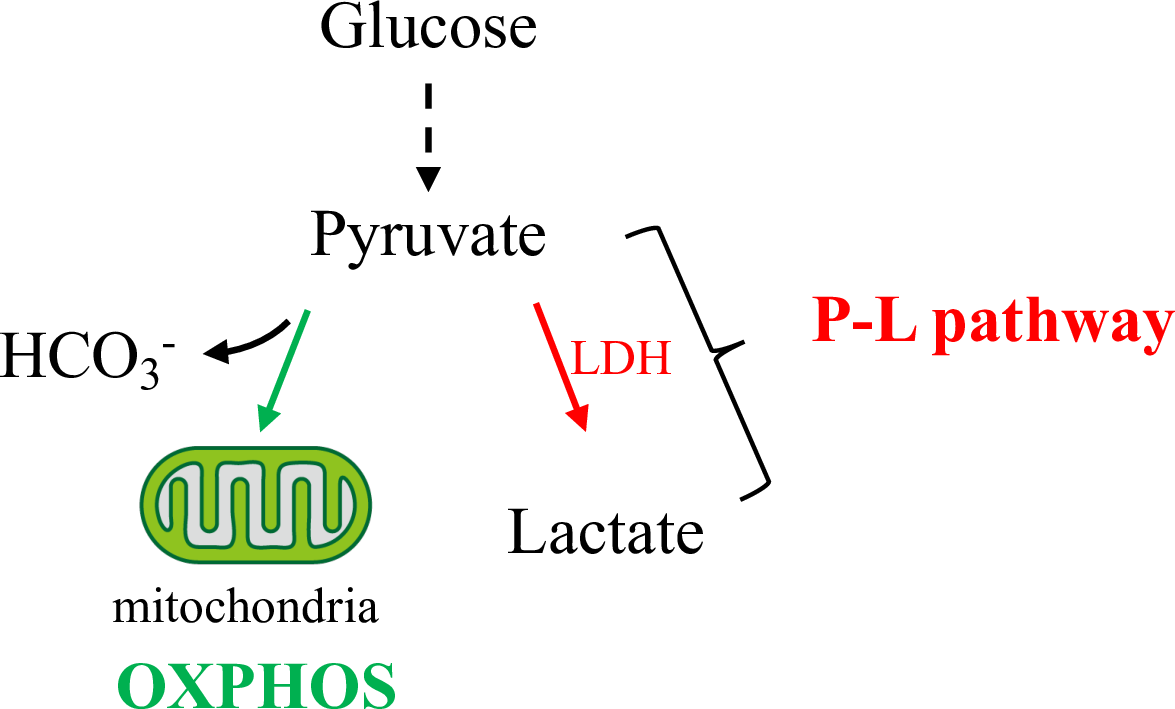

Supplement: S1 Fig — OXPHOS, oxidative phosphorylation; LDH, lactate dehydrogenase. (TIF) [file pone.0309700.s001.tif]

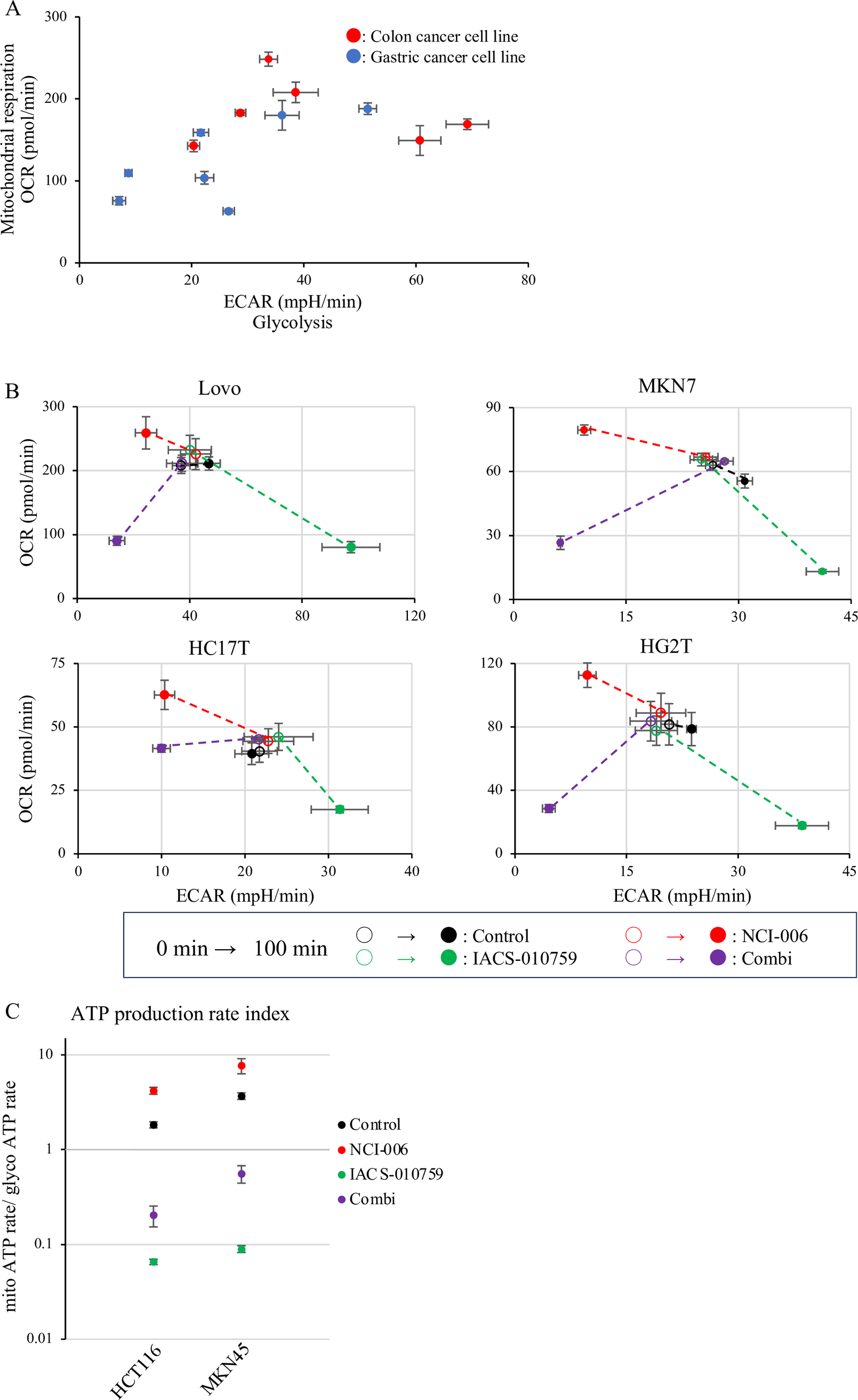

Supplement: S2 Fig — (A)The cell-energy phenotype of each cancer cell line. The cell-energy phenotype was generated based on the OCR/ECAR levels. Data are presented as mean ± SD. (B)Metabolic changes in colorectal and gastric cancer cell lines induced by each inhibitor. Metabolic activity of each cancer cell line was determined based on the OCR/ECAR levels of Lovo, MKN7, HC17T, and HG2T cells with or without treatment. Dashed lines connect the baseline activity (0 min; open symbols) and metabolic activity after treatment (100 min; closed symbols). IACS-010759 at 2 μM and/or NCI-006 at 5 μM were applied. Data are presented as mean ± SD. (C)Inhibitor-induced changes in the production source of ATP. The ATP production rate index was calculated as the ATP value from OXPHOS divided by the ATP value from glycolysis. Data are presented as the mean ± SD. OCR, oxygen consumption rate. (TIF) [file pone.0309700.s002.tif]

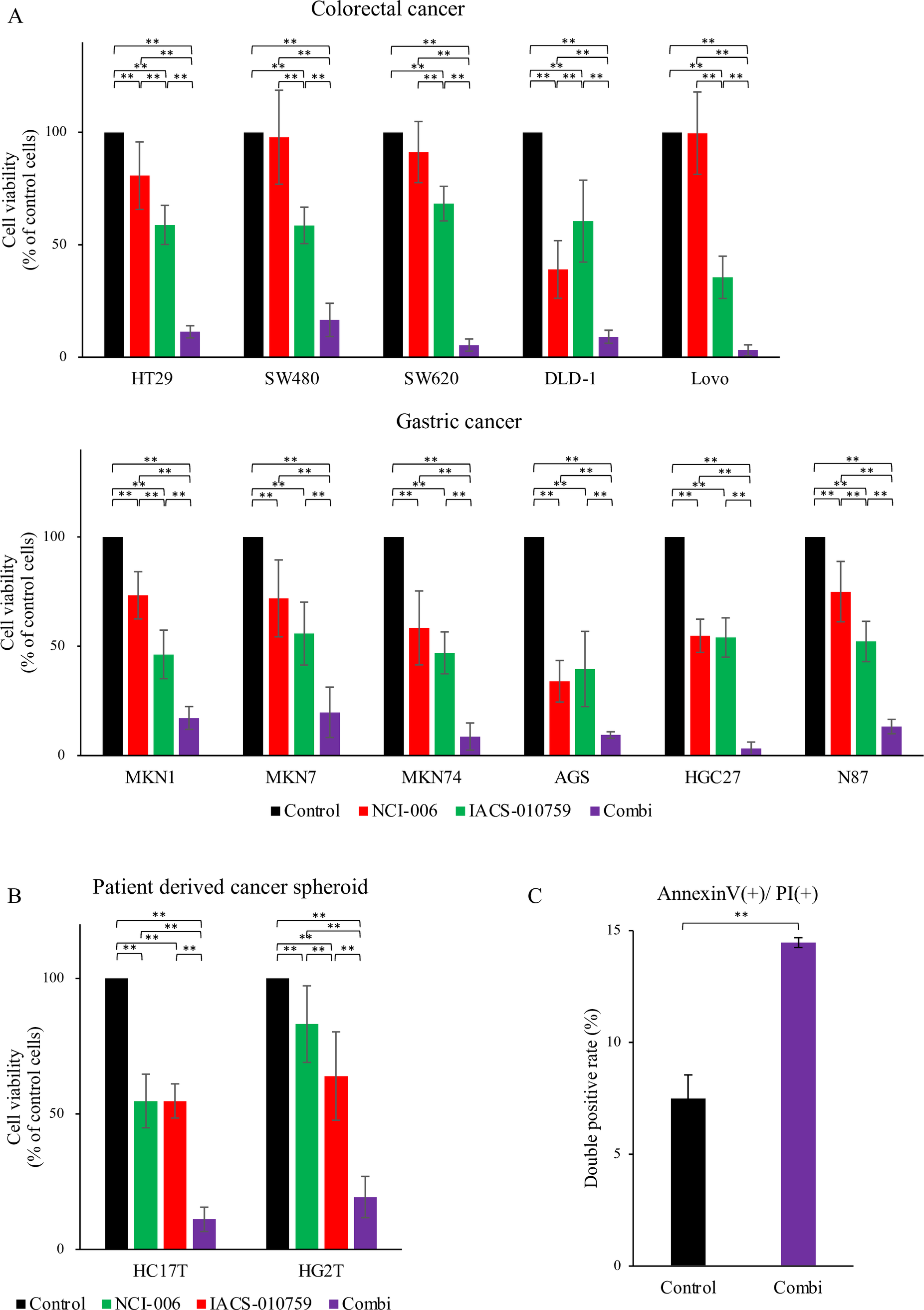

Supplement: S3 Fig — (A)Antitumor effects of NCI-006 and IACS-010759 in colorectal and gastric cancer cell lines. Colorectal and gastric cancer cells were treated with NCI-006 (1 μM) and/or IACS-010759 (1 μM) for 48 h, and cell proliferation was assessed. Data are displayed as mean ± SD (n = 18 for each group; **p < 0.01, two-way ANOVA Tukey test). (B)Antitumor effects of NCI-006 and IACS-010759 in patient-derived cancer spheroids from colon (HC17T) and gastric cancer (HG2T) cells. HC17T and HG2T cells were treated with NCI-006 (1 μM) and/or IACS-010759 (1 μM) for 48 h, and cell proliferation was assessed. Data are displayed as mean ± SD (n = 18 for each group; **p < 0.01, two-way ANOVA Tukey test). HC17T, patient-derived colon cancer spheroids; HG2T, patient-derived gastric cancer spheroids. (C)Flow cytometric evaluation of apoptosis in HCT116 cells. The rate of apoptotic cells was evaluated using Annexin V and propidium iodide staining and compared with vehicle and combined treatment (1 μM NCI-006 + 1 μM IACS-010759) for 24 h. Data are displayed as mean ± SEM (n = 3, ** p < 0.01). (TIF) [file pone.0309700.s003.tif]

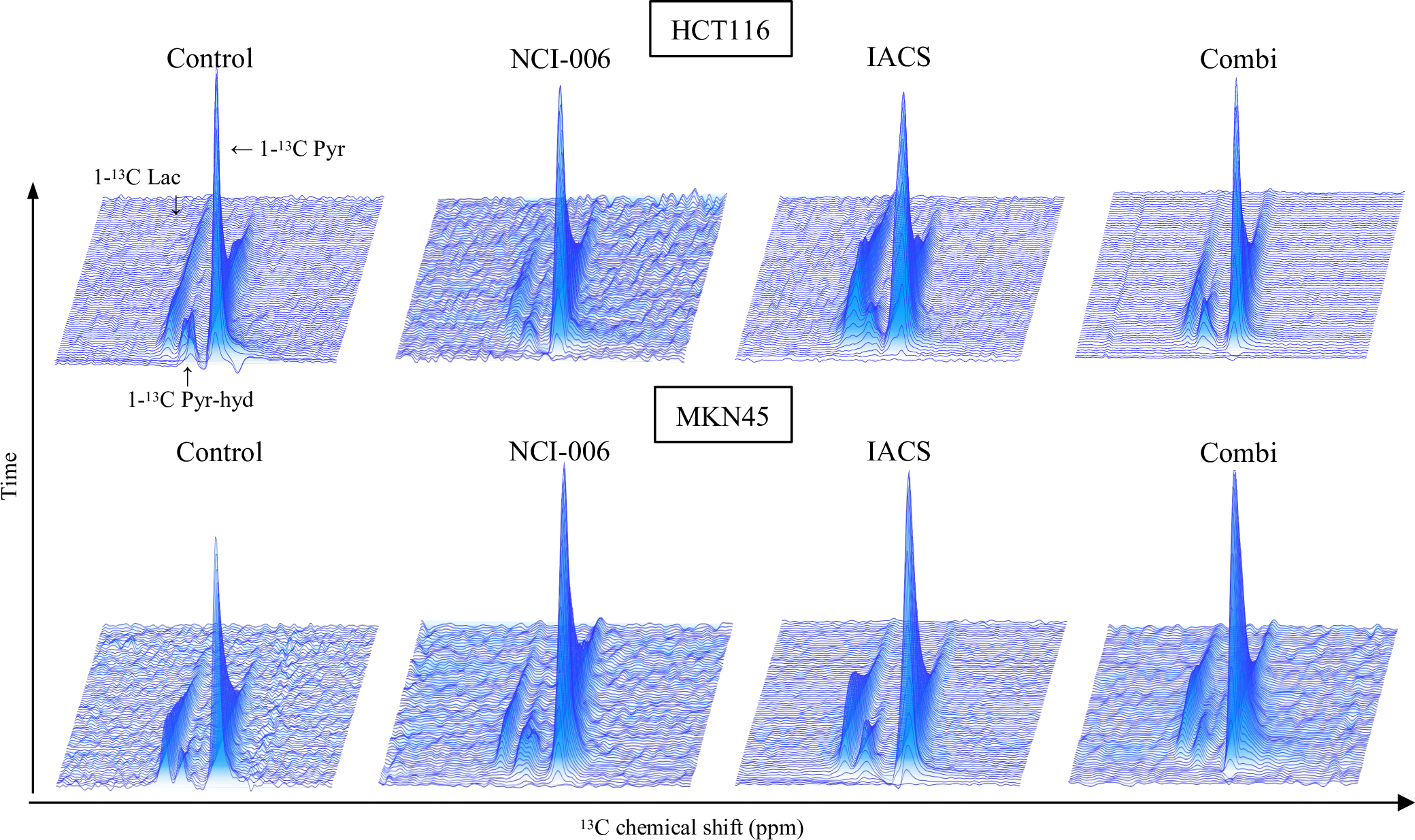

Supplement: S4 Fig — The results correspond to Fig 4C. (TIF) [file pone.0309700.s004.tif]

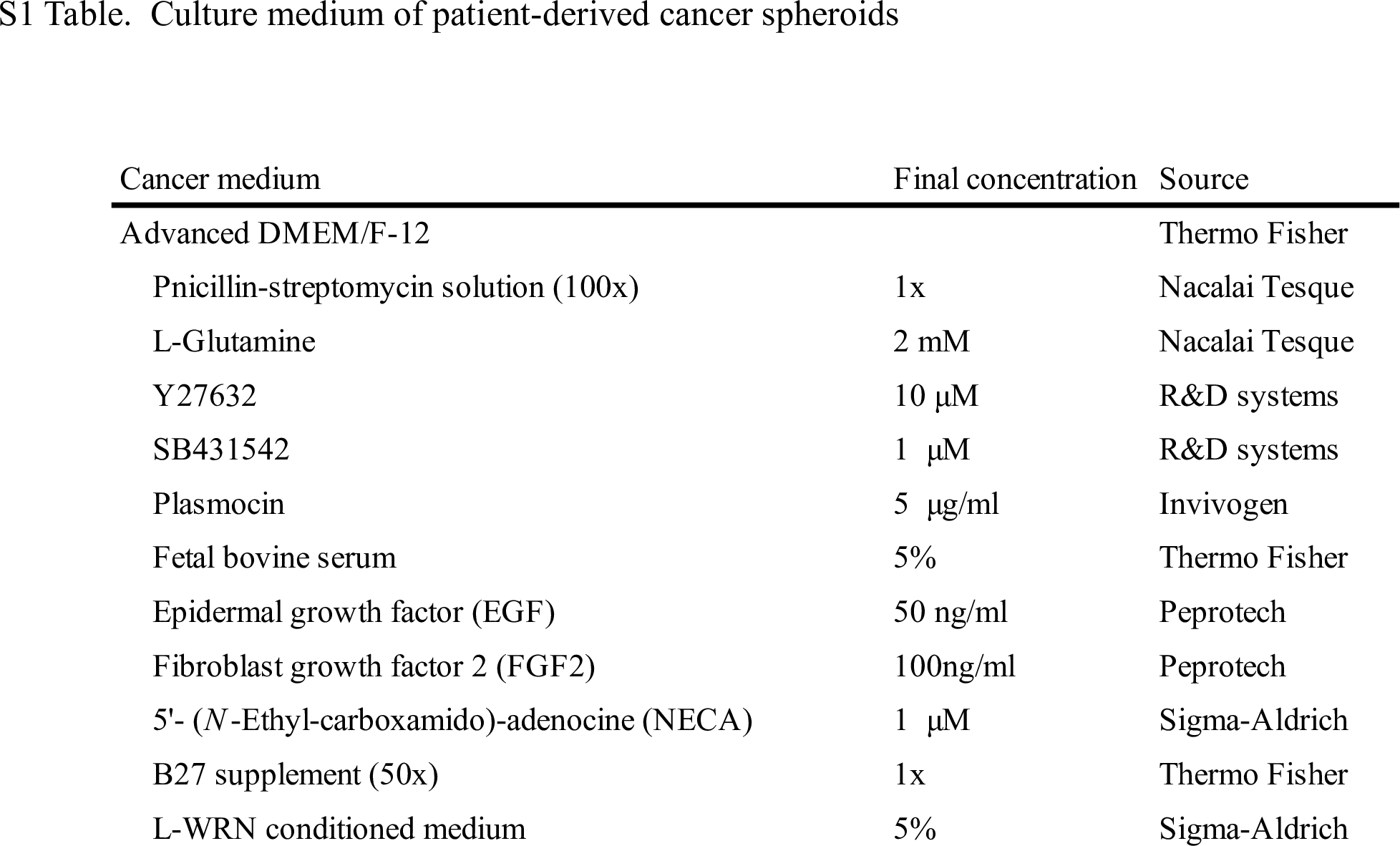

Supplement: S1 Table — (TIF) [file pone.0309700.s005.tif]

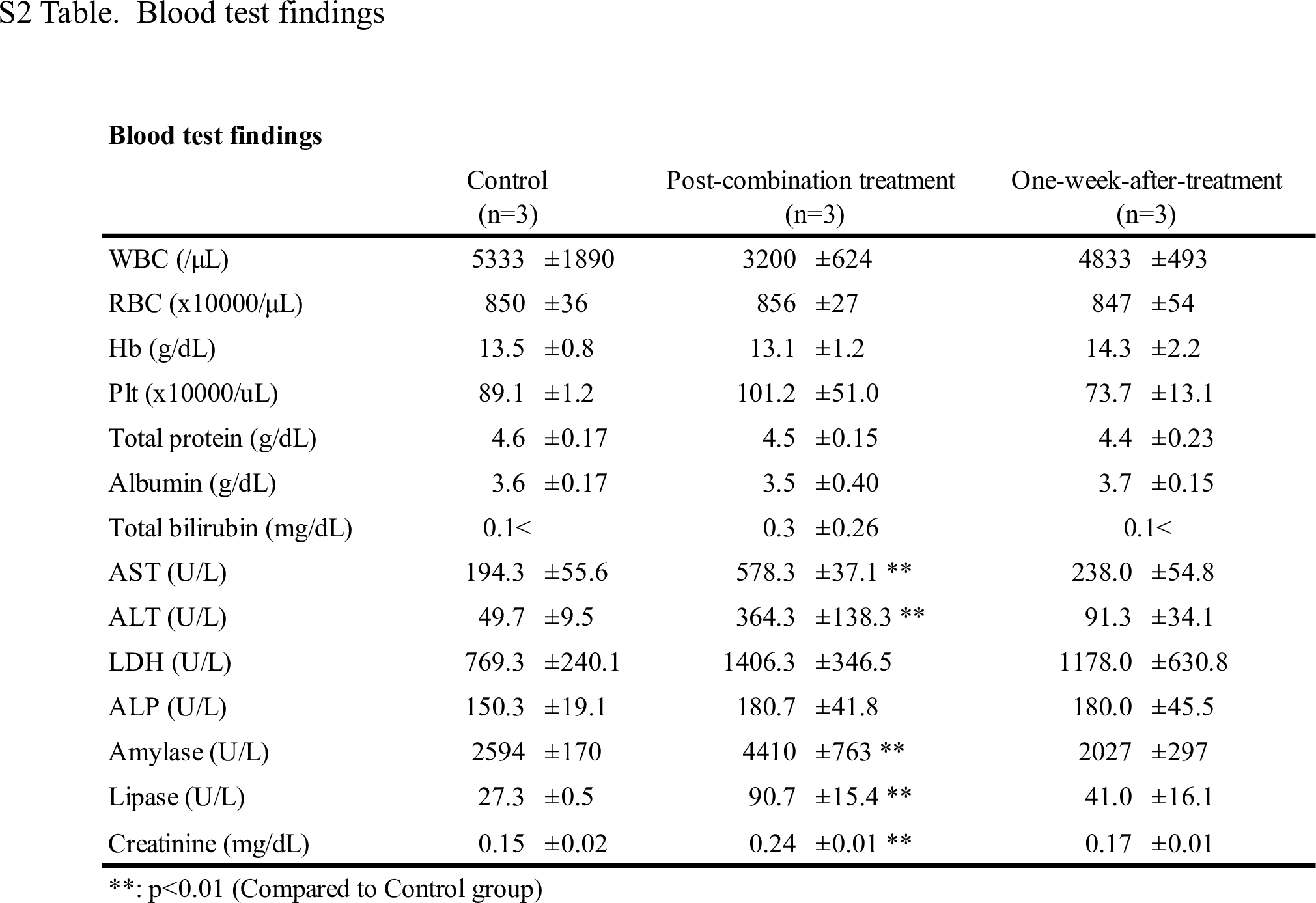

Supplement: S2 Table — (TIF) [file pone.0309700.s006.tif]
